# Multimodal intervention in older adults improves resting-state functional connectivity between the medial prefrontal cortex and medial temporal lobe^†^

**DOI:** 10.3389/fnagi.2014.00039

**Published:** 2014-03-10

**Authors:** Rui Li, Xinyi Zhu, Shufei Yin, Yanan Niu, Zhiwei Zheng, Xin Huang, Baoxi Wang, Juan Li

**Affiliations:** ^1^Center on Aging Psychology, Key Laboratory of Mental Health, Institute of Psychology, Chinese Academy of SciencesBeijing, China; ^2^Magnetic Resonance Imaging Research Center, Institute of Psychology, Chinese Academy of SciencesBeijing, China

**Keywords:** intervention, plasticity, aging, fMRI, functional connectivity

## Abstract

The prefrontal cortex and medial temporal lobe are particularly vulnerable to the effects of aging. The disconnection between them is suggested to be an important cause of cognitive decline in normal aging. Here, using multimodal intervention training, we investigated the functional plasticity in resting-state connectivity of these two regions in older adults. The multimodal intervention, comprised of cognitive training, Tai Chi exercise, and group counseling, was conducted to explore the regional connectivity changes in the default-mode network, as well as changes in prefrontal-based voxel-wise connectivity in the whole brain. Results showed that the intervention selectively affected resting-state functional connectivity between the medial prefrontal cortex and medial temporal lobe. Moreover, the strength of resting-state functional connectivity between these regions correlated with individual cognitive performance. Our results suggest that multimodal intervention could postpone the effects of aging and improve the function of the regions that are most heavily influenced by aging, as well as play an important role in preserving the brain and cognition during old age.

## INTRODUCTION

Although normal aging causes changes in brain anatomy and function that result in decreased cognitive performance (Andrews-Hanna et al., 2007; Tomasi and Volkow, 2012), neural plasticity is preserved in the aging brain (Burke and Barnes, 2006). Increasing evidence suggests that interventions promoting more involvement in activities that are cognitively, socially, and physically stimulating may help optimize structural morphology and cerebral functions (Boyke et al., 2008; Voss et al., 2010; Erickson et al., 2011; Pieramico et al., 2012). Furthermore, such interventions are beneficial for maintaining a healthy brain and reducing the risk of Alzheimer’s disease in the elderly (Nyberg et al., 2012).

The changes occurring in the brain during normal aging are heterogeneous and selective (Burke and Barnes, 2006). Neuroimaging and cellular connectivity studies have generally shown that aging can greatly influence the structural integrity of the frontal cortex and medial temporal lobe (MTL); however, aging has progressively lesser influence on the occipital, parietal, and other posterior cortical regions (Ball, 1977; Uylings and de Brabander, 2002; Raz et al., 2004; Voss et al., 2010). Thus, many important cognitive functions that depend on the frontal cortex and MTL, such as executive control, memory, and learning, show substantial age-related deficits (Rhodes, 2004; Burke and Barnes, 2006; Cabeza and Dennis, 2012). The frontal cortex is one of the regions which exhibit the greatest age-related neuronal loss (de Brabander et al., 1998) and volumetric declines (Cabeza and Dennis, 2012) in morphology. With aging, functional brain activation in the frontal cortex is reduced, and this region is usually over recruited by older individuals with higher cognitive performance (Grady et al., 2006; Nyberg et al., 2010). Moreover, a study showed that older adults who attended an 8-week method of loci training demonstrated increased cortical thickness in the prefrontal cortex, and the structural change was associated with improvement in memory performance (Engvig et al., 2010). The MTL also shows accelerated volume loss during aging (Raz et al., 2004; Fjell et al., 2013) and is known to be the initial site of histopathological changes in Alzheimer’s disease (Greicius et al., 2004). Evidence from a recent longitudinal study suggests that in older adults free of dementia, the MTL shows the greatest volumetric reductions, followed by the prefrontal cortex (Fjell et al., 2013). Further, the structural and functional changes in the MTL have been linked to cognitive decline in older adults (Persson et al., 2012). Thus, in view of the crucial roles that the frontal cortex and MTL play in aging, interventions aimed at improving the functions of these regions would be especially beneficial for countering the age-related decline observed in older individuals.

Various interventions and training programs, such as cognitive training (Engvig et al., 2010; Kirchhoff et al., 2012; Lovden et al., 2012) and physical training (Colcombe et al., 2004; Voss et al., 2010), are being introduced to investigate the structural and functional aspects of brain plasticity in older adults. It is generally known that cognitive aging is the combined effect of multiple factors (Nyberg et al., 2012). Some behavioral evidence has indicated that combining cognitive and physical training may enhance the cognitive or physical abilities of older adults more than either of the training programs would alone (Fabre et al., 2002; Oswald et al., 2006). This evidence supports the idea that a multimodal intervention program that integrates a variety of cognitive, physical, and social activities would be a more conducive way to improve brain function in older adults.

Here, we developed a multimodal intervention program composed of cognitive training, Tai Chi exercise, and group counseling to measure the functional plasticity of the frontal cortex and MTL in older adults using functional magnetic resonance imaging (fMRI). In particular, we focused on the default-mode network (DMN). The DMN involves the frontal cortex and MTL (Greicius et al., 2003; Buckner et al., 2008; Whitfield-Gabrieli and Ford, 2012), and is functionally relevant to internal mental explorations (self-referential thought, remembering, planning, and autobiographical memory), episodic memory, and other related cognitive functions (Greicius et al., 2003, 2004; Buckner and Carroll, 2007). Neuroimaging studies have consistently reported a strong association between the aging process and disruptions in DMN connectivity (Andrews-Hanna et al., 2007; Mevel et al., 2011). Moreover, recent evidence from the cognitive reserve field has suggested that stimulating activities can modify the functional activity and connectivity in the DMN (Bosch et al., 2010; Bastin et al., 2012; Arenaza-Urquijo et al., 2013). In the present study, we examined whether multimodal intervention training helps older individuals improve their brain function.

Because of the aforementioned vulnerability of the frontal and MTL regions caused by aging and the involvement of these regions in critical cognitive functions, we were especially interested in identifying whether the intervention could improve the functional connectivity between the medial prefrontal cortex (mPFC) and MTL regions (hippocampal formation, HF and parahippocampal cortex, PHC) in the DMN. Additionally, we aimed to determine whether the improved mPFC-MTL connectivity would help the elderly achieve higher scores on relevant functional tests. To test these hypotheses, we first examined whether the functional connectivity between the mPFC and MTL regions showed plasticity after intervention. Then, we validated the specificity of the functional plasticity of the mPFC-MTL connectivity by evaluating the connectivity of additional regions in the DMN and of the whole brain. Lastly, we investigated the relationship between fMRI connectivity in the brain and individual performance as measured by a set of neuropsychological tests.

## MATERIALS AND METHODS

### PARTICIPANTS

**FIGURE 1:**
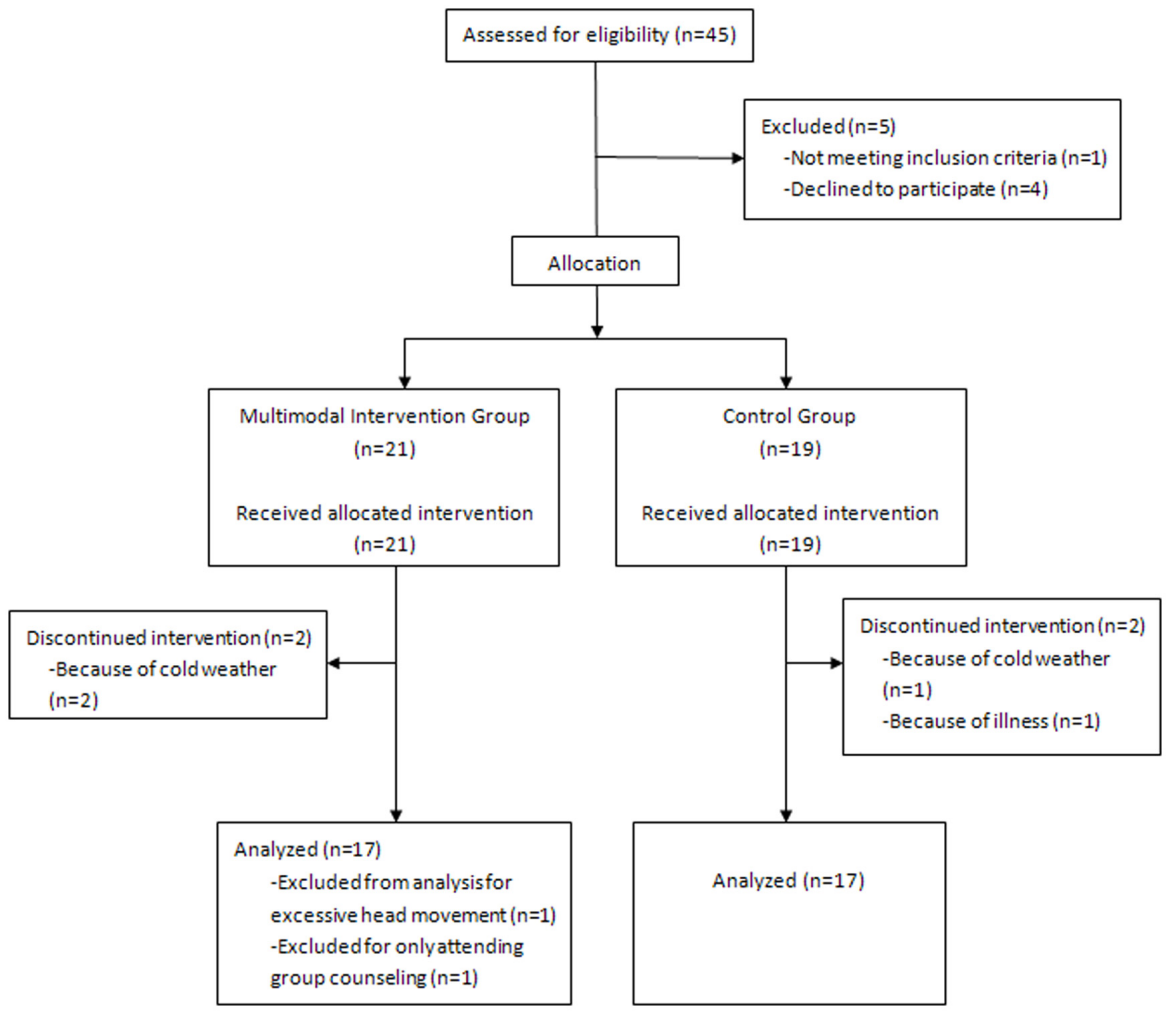
**The flow chart of recruitment and exclusion of participants**.

For this study, we recruited 45 healthy older volunteers (26 in the intervention group and 19 in the control group) residing in two communities near the Institute of Psychology at the Chinese Academy of Sciences. The participants were recruited through advertisements posted at the community service stations. After baseline evaluation, two communities were randomly allocated to the intervention or control group. This allocation was made to keep participants blind to the study design. All participants met the following inclusion criteria: (1) age ≥60 years; (2) education ≥6 years; (3) a score of ≥21 on the Beijing Version of the montreal cognitive assessment (MoCA; Yu et al., 2012); (4) a score of <16 on the Center for Epidemiologic Depression Scale (CES-D; Roberts and Vernon, 1983); (5) right-handed; (6) activities of daily living (ADL) score of ≤16 (Lawton and Brody, 1969); and (7) free of neurological and psychiatric disorders and traumatic brain injury. Based on these criteria, 11 subjects were excluded from further analyses. As shown in the flow chart (**Figure 1**), this included one participant with an education level of only 3 years, four participants who declined to participate in the intervention because of schedule conflicts after the baseline evaluation and before the intervention began, four participants who dropped out during the intervention because of cold weather or illness, one participant in the intervention group who only attended group counseling, and one subject with excessive head movement during scanning. 34 participants were included in the final analyses: 17 in the intervention group (9 men, mean age: 68.6 ± 5.7 years) and 17 in the control group (11 men, mean age: 71.7 ± 4.0 years).

Participants completed a battery of standardized assessments and MRI scanning individually before and after the interventions. The baseline assessment and scan for two groups were conducted in 2 weeks before the intervention began. Both groups did not receive post-intervention assessment and scan until the intervention was over, and the assessment and scan were completed in 2 weeks as the baseline evaluation.

A standardized battery of tests was administered to each participant to evaluate the effects of the intervention on cognitive function, health status, social support, and subjective well-being. Tests of cognitive function included the Paired Associative Learning Test (PALT; Xu and Wu, 1986), digit span forward and digit span backward (Gong, 1992), Trail Making Test (TMT; Reitan, 1986), Stroop Test (Stroop, 1935), and Category Fluency Test (CFT; Spreen and Strauss, 1998). Health status was measured by the Medical Outcomes Study Short Form-36 (MOS SF-36; Ware, 2000). The level of social support was measured by the Social Support Rating Scale (SSRS; Xiao, 1994). Subjective well-being was measured by the Satisfaction with Life Scale (SWLS; Diener et al., 1985) and the Index of Well-Being (IWB; Campbell et al., 1976). The examiners who conducted the test battery were blind to the status of participants (intervention or control).

This study was approved by the institutional review board of the Institute of Psychology, Chinese Academy of Sciences. All participants provided written informed consent before taking part in our experiments.

### INTERVENTION PROGRAM

The intervention was composed of multimodal activities including cognitive intervention, Tai Chi exercise, and group counseling that were carried out over a 6-week period. Subjects in the control group were instructed to attend two 120-min lectures on health and aging.

The cognitive intervention was comprised of two different parts: mnemonic training (MT) and executive function training (EFT). The cognitive intervention consisted of 18 1-h sessions over 6 weeks, which alternated between MT and EFT (nine sessions each). Sessions were conducted in small groups (5–10 persons) three times per week. Each group completed their training sessions under the guidance of an instructor, who followed a detailed training manual. Participants learned a series of mnemonics, including the generation of mental images, associating items (through interactive imagery and sentence generation), and the method of loci. Homework was assigned after each MT session for further home practice of mnemonics. During the EFT sessions, participants played three EFT games on tablets. The three self-developed games were designed to train three components of executive function: inhibition, switching, and updating. Feedback on performance was provided to the subjects.

The Tai Chi exercise consisted of 1-h sessions held three times per week for 6 weeks. During the 18 sessions, an experienced Tai Chi instructor gradually taught the Yang-Style 24-form Tai Chi. Participants were asked to imitate the motions and postures of the instructor. Two additional teaching assistants monitored the practice and assisted in correcting inaccurate postures of the participants. At the end of 6 weeks, all participants had learned the entire sequence.

Group counseling was aimed at promoting the psychological well-being of older adults through reminiscence. Counseling was provided in small groups of 8–10 participants. The counseling sessions were held weekly for 6 weeks and led by licensed counseling psychologists. The six 90-min sessions focused on three different topics: career, family, and health, with each topic lasting for two sessions. Group members were encouraged to review life histories and to share experiences related to the session topic. Counseling emphasized the process of reviewing and interpreting positive experiences from one’s past and finding the sense of self and meaning of life. Interpersonal communications and the development of friendships were also encouraged. Homework activities were assigned after each session to encourage intrapersonal reminiscence and to help prepare participants for the next session.

### STATISTICAL ANALYSIS OF NEUROPSYCHOLOGICAL DATA

Baseline characteristics of participants in both the intervention and control group were examined using a two-tailed Pearson’s chi-square test, a two-sample two-tailed Student’s *t*-test, or a non-parametric (Mann–Whitney) test. The intervention effect was investigated by conducting a repeated measures two-way analysis of variance (ANOVA). Individual performance on each neuropsychological test at baseline and post-intervention was the within-subjects factor, while group performance was the between-subjects factor. All statistical analyses were conducted using SPSS 19.0 (IBM Corporation, Somers, NY, USA).

### IMAGE ACQUISITION

Both the pre- and post-intervention scans were performed on each of the 34 subjects using the same fMRI protocols. Images from all subjects were collected using a 3.0-Tesla Siemens Trio scanner (Erlangen, Germany) located at the Beijing MRI Center for Brain Research. During each scan, participants were asked to relax, close their eyes, and keep their head still without falling asleep. For each participant, functional images were collected using an echo-planar imaging sequence with the following parameters: repetition time (TR) = 2000 ms; echo time (TE) = 30 ms; flip angle = 90°; field of view (FOV) = 200 mm × 200 mm; slice thickness = 3.0 mm; gap = 0.6 mm; acquisition matrix = 64 × 64; in-plane resolution = 3.125 × 3.125; 33 axial slices; and 200 volumes. A high-resolution structural T1-weighted magnetization-prepared rapid gradient-echo image was also collected for each subject with the following parameters: 176 slices; acquisition matrix = 256 × 256; voxel size = 1 mm × 1 mm × 1 mm; TR = 1900 ms; TE = 2.2 ms; flip angle = 9°.

### IMAGE PROCESSING AND ANALYSIS

Data pre-processing was performed using the Statistical Parametric Mapping program (SPM8^1^), Resting-State fMRI Data Analysis Toolkit (REST V1.8^2^), and the Data Processing Assistant for Resting State fMRI (DPARSF V2.2^3^). The first five volumes were discarded to allow for equilibration of the magnetic field and adaptation of the subjects to the scanning environment. The remaining 195 volumes were corrected for intra-volume acquisition time differences between slices using the Sinc interpolation and were corrected for inter-volume geometrical displacement due to head motion using a six-parameter (rigid body) spatial transformation. No participant included in this study exhibited head motion of more than 2.0 mm maximum translation and 2.0° rotation throughout the pre-training and post-training scans. The functional images were then normalized to the standard Montreal Neurological Institute (MNI) space (resampling voxel size, 3 mm × 3 mm × 3 mm), and were spatially smoothed with a 4-mm full width at half maximum Gaussian kernel to decrease spatial noise. Finally, detrending and temporal band-pass filtering (0.01–0.08 Hz) was performed to reduce the effects of low-frequency drifts and high-frequency physiological noise. We also regressed out several sources of artifacts including the six head-motion profiles, global signal, white matter signal, and cerebrospinal fluid signal. The residual volumes were retained for use in the connectivity analysis.

**FIGURE 2:**
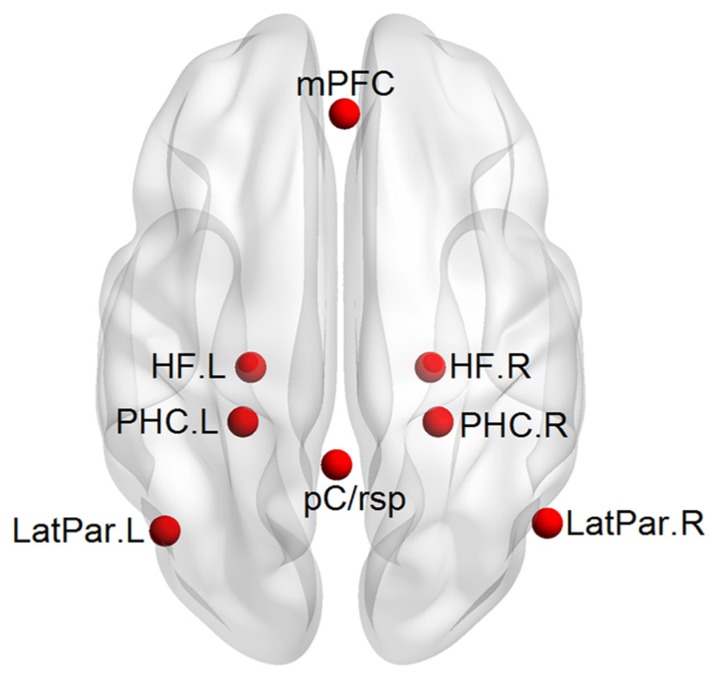
**An axial view of the 8 ROIs defined in the DMN**.

*A priori* mPFC-MTL connectivity analysis: a hypothesis-driven analysis in which the functional connectivity between the mPFC and MTL would be improved after the multimodal intervention was firstly performed. Five regions of interest (ROIs) including the mPFC, bilateral HF, and bilateral PHC were selected *a priori* in the DMN by referring to those previously used by Andrews-Hanna et al. (2007; **Figure 2**). Each ROI was defined as a 6-mm spherical region. The center coordinates in the atlas of Talairach-Tournoux were as follows: mPFC (1, 40, 16), left and right HF [HF.L, (-23, -25, -12) and HF.R, (23, -25, -12)], and left and right PHC [PHC.L, (-25, -39, -10) and PHC.R, (25, -39, -10)]. We extracted the mean time series of each ROI for each subject and then calculated the Pearson’s correlation coefficient (*r*) between the time series of mPFC and each of the other four MTL regions. The correlation coefficient (*r*) represents the strength of the functional connectivity between regions. A Fisher *r*-to-*z* transformation was performed for the correlation coefficients (*r*) to generate normalized functional connectivity *z*(*r*). To evaluate the intervention effect on functional connectivity between the mPFC and MTL, a Group (control, intervention, between-subjects) × Intervention (pre, post, within-subjects) ANOVA was performed for each of the four connectivity pairs (*p *< 0.05, Bonferroni corrected), with age, gender, and education level as covariates. Particularly, individual baseline connectivity differences were controlled for in the interactional analysis. We then performed paired sample *t*-tests (*p *< 0.05) to evaluate the changes in the connectivity with the Group × Intervention interactions for each group. The ANOVA and paired sample *t*-tests in the current study were all performed using SPSS 19.0 (IBM Corporation, Somers, NY, USA).

Validating the functional plasticity of the mPFC-MTL connectivity: to examine the specificity effect of the intervention on the functional connectivity between the mPFC and MTL, we conducted an additional region-to-region correlation analysis and a whole-brain exploratory analysis. First, three additional DMN regions including the posterior cingulate/retrosplenial cortex [pC/rsp, (-1, -50, 26)] and left and right lateral parietal cortices [LatPar.L, (-45, -67, 26) and LatPar.R, (53, -65, 26)] were defined. They were combined with the mPFC and MTL regions for analyzing the intervention effect on the additional 24 connectivity pairs. The calculation of interregional correlation and the statistical analysis were the same as the above *a priori* connectivity analysis. Second, we employed the mPFC as a seed region and generated the whole-brain correlation map to evaluate further the specificity of the mPFC-MTL plasticity. The mPFC is a major component in the DMN, and some previous studies have used the mPFC to generate the DMN pattern from resting-state fMRI data (Andrews-Hanna et al., 2007). We calculated the Pearson’s correlation coefficient (*r*) between the time course of the seed and the time series of each voxel across the whole brain. After a Fisher’s* r*-to-*z* transformation of correlation coefficient maps to *z* maps, a voxel-wise one-sample *t*-test (*p* < 0.05, AlphaSim corrected) was performed to generate the functional connectivity map of the mPFC, both before and after intervention for both groups. Finally we did a Group (control, intervention, between-subjects) × Intervention (pre, post, within-subjects) ANOVA (*p* < 0.05, AlphaSim corrected) to identify regions that showed training-related interactions in functional connectivity with the seed. Paired sample *t*-tests were also performed to evaluate the changes in these regions showing interactional effects.

Connectivity-behavioral analysis: multiple correlational analyses (*p* < 0.05, Bonferroni corrected) were performed to evaluate the relationship between fMRI connectivity and individual performance.

## RESULTS

### BASELINE DEMOGRAPHICAL AND CLINICAL CHARACTERISTICS

**Table 1** shows the demographical and clinical characteristics of the participants. The intervention and control groups did not differ significantly in gender, age, years of education, or on MoCA, CES-D, and ADL scores.

**Table 1:** Demographics and neuropsychological characteristics of the participants.

Characteristic	Intervention group	Control group	*p*
(M/F)	17 (9/8)	17 (11/6)	0.50^a^
Age, years	68.6 ± 5.7	71.7 ± 4.0	0.08^b^
Education, years	13.3 ± 3.1	14.7 ± 3.3	0.22^b^
MoCA	26.3 ± 2.7	25.2 ± 2.5	0.33^b^
CES-D	7.0 ± 5.5	6.8 ± 6.1	0.85^c^
ADL	14.1 ± 0.5	14.2 ± 0.7	0.97^c^

a The *p *value was obtained using a two-tail Pearson chi-square test.

b The *p* value was obtained using a two-sample two-tail *t*-test.

c The *p* value was obtained using non-parametric (Mann–Whitney) test.

### NEUROPSYCHOLOGICAL MEASURES OF INTERVENTION EFFECTS

Analysis of the effects of intervention on outcome measures revealed significant Group × Intervention interactions (*p *< 0.05) for PALT, SSRS, and physical vitality. Improvement on PALT occurred in the trained group (*p *= 0.028), but not in the control group (*p *= 0.506). Participants in both the intervention group (*p *< 0.001) and the control group (*p *= 0.007) showed significant increases in SSRS levels, but the change in the intervention group was larger than that in the control group. After the intervention, physical vitality was improved in the intervention group (*p *= 0.057), but did not change in the control group (*p *= 0.255). In addition, there was a trend for Group × Intervention interactions on the TMT (*p *= 0.055) and SWLS (*p *= 0.072). Further analysis revealed that performance on the TMT and SWLS was unchanged in the trained group (*p *= 0.855 and 0.831, respectively), but declined in the control group (*p* = 0.013 and 0.022, respectively).

### *A PRIORI* mPFC-MTL CONNECTIVITY ANALYSIS

**FIGURE 3:**
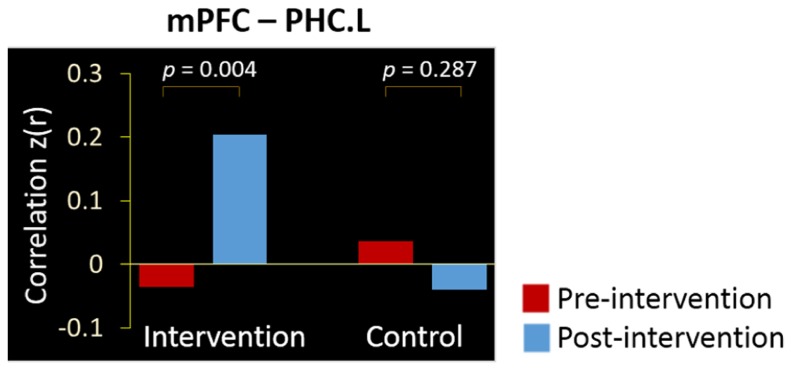
**Intervention positively affected the resting-state connectivity between the mPFC and PHC.L.** Bars denote the mean correlations *z*(*r*) between the regions in intervention and control groups at the pre-intervention scan (red) and post-intervention scan (blue).

We calculated the regional connectivity between the mPFC and four MTL regions in the DMN, and applied a Group (control, intervention, between-subjects) × Intervention (pre, post, within-subjects) ANOVA to examine the intervention effect on each of the four mPFC-MTL connectivity pairs (Bonferroni corrected for four comparisons, threshold at 0.05/4 = 0.0125). Results revealed a significant Group × Intervention interaction for the connectivity between the mPFC and PHC.L (*p* = 0.001). As shown in **Figure 3**, the intervention group showed a dramatic increment in functional correlation between the two regions after the training activities (from *z*(*r*) = -0.036 to 0.201, *p *= 0.004), while in the control group, there was no significant change in the mPFC-PHC.L connectivity between the two scans (from *z*(*r*) = 0.036 to -0.040, *p *= 0.287).

### VALIDATION OF THE SPECIFICITY OF mPFC-MTL PLASTICITY

When examining the other 24 connectivity pairs in the DMN, we did not observe any significant Group × Intervention interactions for any of the connectivity pairs (Bonferroni correction threshold at 0.05/24 = 0.0021). **Table 2** details the correlation strength between the DMN regions at pre- and post-intervention and the Group × Intervention ANOVA result for each connectivity pair.

**Table 2:** Correlation coefficients *z*(*r*) between DMN regions.

	Connectivity	Intervention (*n *= 17)		Control (*n *= 17)		*p*
		Pre-intervention	Post-intervention	Pre-intervention	Post-intervention
mPFC-MTL connectivity	mPFC-HF.L	-0.068	0.080	0.031	0.031	0.153
	mPFC-HF.R	0.056	0.076	0.021	0.081	0.737
	mPFC-PHC.L*	-0.036	0.201	0.036	-0.040	0.001
	mPFC-PHC.R	-0.049	0.047	-0.015	-0.060	0.099
Other connectivity in the DMN	mPFC-pC/rsp	0.113	0.082	0.071	0.153	0.316
	mPFC-LatPar.L	0.063	-0.015	0.063	0.161	0.032
	mPFC-LatPar.R	0.069	0.072	0.031	0.106	0.554
	pC/rsp-LatPar.L	0.591	0.647	0.550	0.597	0.644
	pC/rsp-LatPar.R	0.594	0.532	0.462	0.446	0.930
	pC/rsp-HF.L	0.048	0.146	-0.055	-0.103	0.005
	pC/rsp-HF.R	0.021	-0.005	-0.038	-0.048	0.934
	pC/rsp-PHC.L	0.204	0.208	0.050	0.007	0.383
	pC/rsp-PHC.R	0.117	0.081	-0.158	-0.089	0.752
	LatPar.L-LatPar.R	0.689	0.645	0.607	0.503	0.537
	LatPar.L-HF.L	0.086	0.128	0.428	0.008	0.085
	LatPar.L-HF.R	0.048	0.023	0.053	0.048	0.728
	LatPar.L-PHC.L	0.125	0.156	0.161	0.064	0.423
	LatPar.L-PHC.R	-0.020	-0.031	-0.073	-0.076	0.791
	LatPar.R-HF.L	0.070	0.065	-0.058	0.053	0.941
	LatPar.R-HF.R	0.036	0.080	0.023	0.150	0.147
	LatPar.R-PHC.L	0.155	0.227	0.028	0.067	0.071
	LatPar.R-PHC.R	0.074	0.040	-0.084	0.027	0.572
	HF.L-HF.R	0.279	0.292	0.542	0.556	0.668
	HF.L-PHC.L	0.245	0.148	0.286	0.318	0.211
	HF.L-PHC.R	0.242	0.172	0.238	0.185	0.838
	HF.R-PHC.L	0.155	0.147	0.394	0.285	0.450
	HF.R-PHC.R	0.181	0.268	0.347	0.269	0.703
	PHC.L-PHC.R	0.477	0.388	0.414	0.405	0.361

*Significant Group × Intervention interaction at a Bonferroni corrected threshold of 0.0125.
*

**FIGURE 4:**
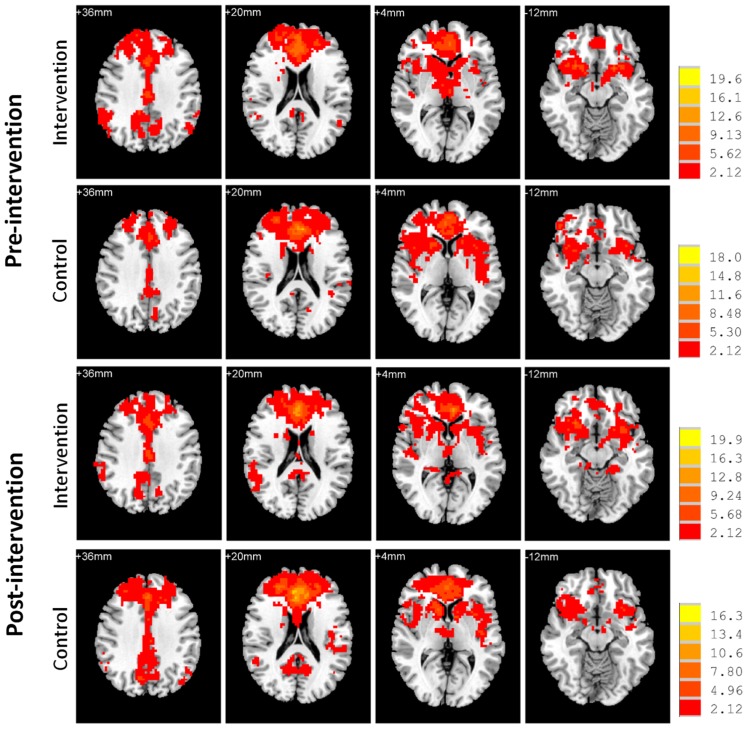
**Functional connectivity map of the mPFC.** Whole-brain analyses of functional correlations between the mPFC and each voxel across the brain are visualized, respectively, for intervention and control groups both before and after intervention (*p* < 0.05, AlphaSim corrected). Bars at the right show *t*-values. Images are in radiologic format with subject left on image right.

**FIGURE 5:**
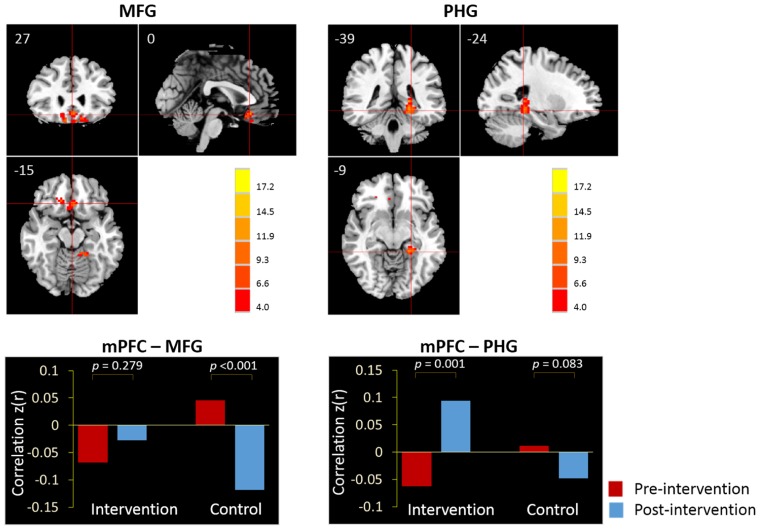
**The whole-brain exploratory analysis of resting-state functional connectivity demonstrated significant Group × Intervention interactions in the MFG and PHG.** The MFG and PHG were, respectively, demonstrated in the sagittal, coronal, and axial views (*p* < 0.05, AlphaSim corrected). Bars at the bottom show the mean correlations *z*(*r*) of mPFC-MFG and mPFC-PHG in intervention and control groups at the pre-intervention scan (red) and post-intervention scan (blue).

Next, we examined the whole-brain functional correlations between the mPFC seed and each voxel across the whole brain. **Figure 4** demonstrates the correlation map of the mPFC both before and after intervention for both groups. The connectivity patterns generated from the mPFC seed included the main areas of the network, but mostly concentrated on the anterior DMN. The analyses of the intervention effect on the functional connectivity of the mPFC found that only two clusters including the medial frontal gyrus (MFG; MNI coordinates: 0, 27, -15; 113 voxels) and the left parahippocampal gyrus (PHG; MNI coordinates: -24, -39, -9; 102 voxels) showed significant Group × Intervention interactions (AlphaSim correction, *p* < 0.05; **Figure 5**). The paired *t*-test performed on connectivity in the two clusters further showed that the intervention group had significantly increased functional connectivity between the seed region and left PHG (from *z*(*r*) = -0.063 to 0.094, *p* = 0.001), while the control group showed significantly decreased connectivity in the MFG (from *z*(*r*) = 0.046 to -0.119, *p* < 0.001; **Figure 5**). As expected, all of the regions showing intervention-related changes were from the prefrontal and medial temporal regions.

### CORRELATION BETWEEN TRAINING-RELATED CONNECTIVITY AND PERFORMANCE

Correlations were calculated between the fMRI connectivity measures and neuropsychological test scores on cognition (TMT, PALT, and CFT), social support (SSRS), subjective well-being (SWLS), and physical vitality.

**FIGURE 6:**
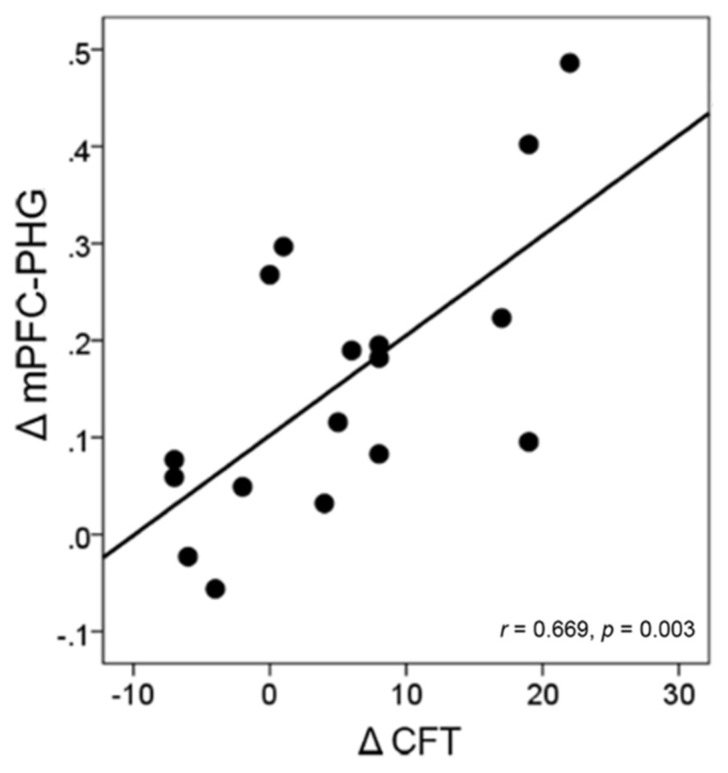
**Correlation between the change in brain connectivity and the change in cognitive performance in the intervention group.** Each circular dot represents the data from one participant. The regression line indicates a positive relationship between the change in the mPFC-PHG connectivity and the change in the performance on CFT.

In the intervention group, we examined the correlations between the changes in the mPFC-MTL connectivity (including the mPFC-PHC.L from *a priori* analyses and the mPFC-PHG from the validation analyses) and the changes in individual performance. The result demonstrated a significant correlation between the changes in the functional connectivity of mPFC-PHG and the changes in cognitive performance (CFT) in the 17 subjects who participated in the intervention activities (*r* = 0.669, *p* = 0.003) at a Bonferroni correction threshold of 0.0042 (0.05/12; **Figure 6**).

**FIGURE 7:**
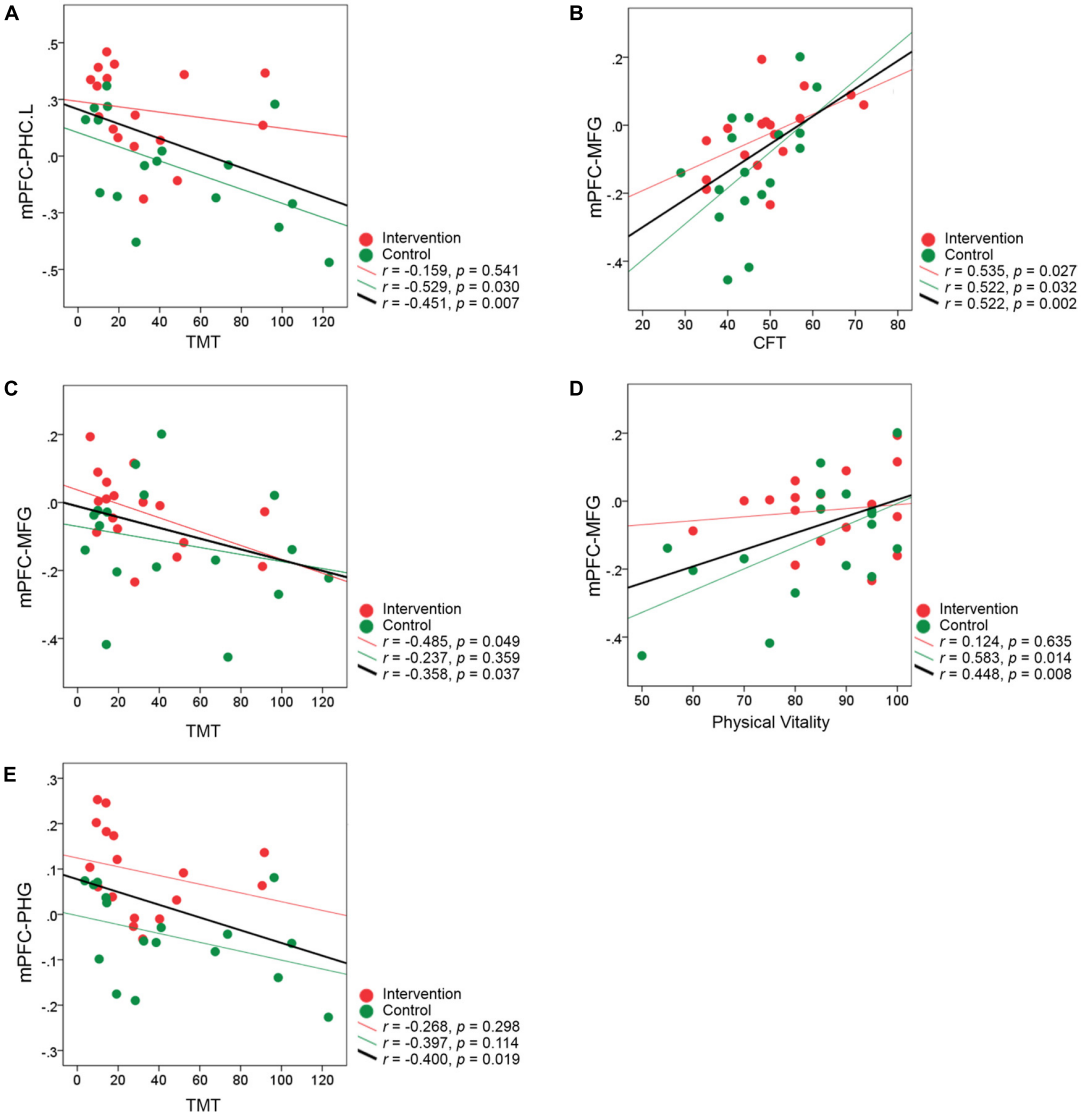
**Correlation between the brain connectivity at the post-intervention scan and individual performance for all subjects. (A–E)** The correlation coefficient *z*(*r*) at the post-intervention scan for each participant is plotted against individual performance after the intervention. Data representing participants from the intervention group are colored in red, and those representing controls are colored in green. The red regression line on each graph represents the relationship between the connectivity and performance in the intervention group, and the green regression line on each graph represents the relationship in the control group. The black regression line indicates the relationship between the post-intervention connectivity and performance for all subjects from the two groups.

In addition, we also explored the relationship between fMRI connectivity at the post-intervention scan and individual performance across all the subjects. The results showed that the level of mPFC-PHC.L connectivity at the post-training scan correlated significantly with individual performance on the TMT (*r *= -0.451, *p* = 0.007) at a Bonferroni-corrected threshold of 0.0083 (0.05/6; **Figure 7A**). Regarding the two clusters including MFG and left PHG from the validation analyses, we also, respectively, calculated the correlations between the connectivity levels and individual performance (Bonferroni correction threshold at 0.05/12 = 0.0042). The results showed that higher functional connectivity levels in the MFG at the post-intervention scan correlated significantly with better performance on the CFT (*r* = 0.522, *p* = 0.002) in all subjects (**Figure 7B**). When a more liberal threshold of *p* < 0.05 without correction was applied, we found that the connectivity level in the MFG also correlated with TMT performance (*r* = -0.358, *p* = 0.037; **Figure 7C**) and physical vitality (*r* = 0.448, *p* = 0.008; **Figure 7D**), and the connectivity level in the left PHG correlated with performance on the TMT (*r* = -0.400, *p* = 0.019; **Figure 7E**). None of the correlations between post-intervention connectivity and individual performance reached significance in either intervention or control groups (all *p*’s > 0.0083).

## DISCUSSION

In the present study, a multimodal intervention trail was performed to investigate the brain plasticity in older adults. The older adults from two similar communities were randomly allocated to two groups: the intervention group receiving a 6-week multimodal intervention that included cognitive training, Tai Chi exercise, and group counseling, and a control group which attended two 120-min lectures on health and aging. By specifically using resting-state fMRI to explore the regional connectivity changes in the DMN as well as changes in prefrontal-based voxel-wise connectivity in the whole brain, we were able to show that the intervention activities improved the resting-state connectivity between the mPFC and MTL regions. Furthermore, we found that the functional connectivity between these two regions was correlated with individual cognitive performance.

Targeted DMN regions and whole-brain exploratory investigations showed that the effects of the intervention on functional connectivity in older adults were rather selective, only influencing the connectivity between the mPFC and certain MTL regions. This result supports our hypothesis on the functional plasticity of the connectivity between the mPFC and MTL. Furthermore, our results suggest that although these regions are impaired by aging, an intervention program could still help strengthen the connectivity between them. Consistent with our findings, previous studies have reported the selective effects of training on these regions. For example, physical training increases the volume of the prefrontal cortex (Colcombe et al., 2006) and hippocampus (Erickson et al., 2011) and can increase hippocampal cerebral blood flow (Burdette et al., 2010) in healthy elderly individuals. The functional activation in the frontal cortex (Kirchhoff et al., 2012) and hippocampus (Belleville et al., 2011) is altered when older adults perform memory tasks. The mechanisms underlying this functional plasticity remain unclear, although some evidence suggests that it could be related to cell proliferation or dendritic growth in the brain after training (Redila and Christie, 2006; Erickson et al., 2011).

A notable difference between our study and former studies is that we investigated the neural plasticity from the point of intrinsic functional cooperation between brain regions. Recent evidence supports the “disconnection” hypothesis of aging, which assumes that the cognitive deficits in older adults emerge from alterations in functionally or structurally coordinated brain regions or systems (O’Sullivan et al., 2001; Pfefferbaum et al., 2005; Salat et al., 2005; Mevel et al., 2011). For example, an fMRI study that assessed the effects of aging on large-scale brain systems demonstrated that functional connectivity in the DMN was severely disrupted with age (Andrews-Hanna et al., 2007). Moreover, impairment in mPFC-HF connectivity was also considered an early sign of Alzheimer’s disease (Wang et al., 2006). Here, the multimodal intervention we developed positively affected the functional connectivity of the DMN in healthy elderly participants. The between-ROIs analysis established that training significantly increased the functional connectivity between the mPFC and left PHC. In the whole-brain exploratory analysis, connectivity between the seed region of the mPFC and left PHG was also significantly increased after training. In contrast, older adults who did not receive intervention training did not improve and even declined on functional connectivity measures. Our results suggest that while control participants exhibited a decline in connectivity due to the aging process, the multi-domain intervention we developed was able to counteract this age-related connectivity decline effectively.

It is important to note that the training-related improvement in functional connectivity was measured by examining the correlations between low-frequency fMRI fluctuations acquired when the participants were at rest (i.e., in the absence of any task). Currently, the functional role of resting-state connectivity within the DMN is not clear (Buckner and Carroll, 2007). Some studies that have characterized the behavioral or cognitive correlates of resting-state functional connectivity suggest that intrinsic connectivity might play a role in the consolidation of previous experiences and in the preparation of future works and could predict individual performance (Lewisa et al., 2009; Tambini et al., 2010; Adelstein et al., 2011; Wei et al., 2012). For example, Albert et al. (2009) and Hasson et al. (2009) demonstrated that resting-state DMN connectivity could be modulated by previous experiences, and Tambini et al. (2010) further demonstrated that modulated resting-state hippocampal-cortical connectivity after an encoding task can predict individual differences in subsequent associative memory performance. In the present study, the series of multi-domain intervention activities were actual events experienced by these older adults, hence there may be some similar or common mechanisms underlying the plasticity of resting-state DMN connectivity. Therefore, although there is no direct task-relevant fMRI connectivity evidence to show whether the older adults can perform tasks better because of improved regional connectivity after effective interventions, the enhanced resting-state mPFC-MTL connectivity potentially reflects an improvement of relevant cognitive and other functions for these older adults.

We investigated the relationship between the functional connectivity observed during the resting-state condition and individual performance on neuropsychological tests. The significant correlation between the change in functional connectivity of the mPFC-PHG and the change in CFT performance in the intervention group (**Figure 6**) suggested that the improved functional coordination between the prefrontal and MTL regions after intervention has benefited the older adults to achieve a higher cognitive performance. When looking into the relationship between fMRI connectivity at the post-intervention scan and individual performance, we found that the resting connectivity level of the mPFC and MTL regions correlated with individual variance in cognitive function (CFT and TMT) and health status (physical vitality) across all subjects from the two groups (**Figure 7**). The CFT is a typical neuropsychological test used to assess language and executive functions, search strategies, and long-term memory (Ruff et al., 1997; Mathuranath et al., 2003; Clark et al., 2009). Many investigations have shown that CFT scores decline greatly with age (Brickman et al., 2005; Clark et al., 2009). Previous studies investigating the neural correlates of CFT have consistently suggested that frontal and medial temporal structures are critical for retrieval by category (Pihlajamaki et al., 2000; Brickman et al., 2005). Our results provide new evidence that the resting-state connectivity between these two important regions could also reflect individual category fluency abilities. The TMT is usually administered to evaluate an individual’s visual attention and task-switching capabilities and can be used to assess executive functions (Sanchez-Cubillo et al., 2009). These abilities are generally presumed to be functions of the frontal lobe (Zakzanis et al., 2005). Recent studies have reported that the decline in TMT performance caused by aging or Alzheimer’s disease correlates with the changes in resting-state DMN connectivity (Schlee et al., 2012; Wang et al., 2013). Additionally, because there are many correlational studies demonstrating the interaction between cognition and physical health status (Huppert, 2006; Thayer et al., 2009), it is not surprising that we observed correlations between resting-state connectivity in the MFG and individual physical vitality. Finally it should be noted that these correlations between the post-intervention connectivity and performance did not reach significance when explored in separate groups. Therefore, although the current results did suggest that the functional connectivity between the prefrontal cortex and MTL was correlate with older adults’ cognitive functions, we could not conclude that the post-intervention connectivity was related to the specific interventions. We speculate that the relatively small number of participants may be a reason to limit us to find significant correlations within single groups.

Several limitations need be noted. First, although we used an active control, the interventions of the two groups were not matched in frequency or duration, which is a major limitation of the present study. Thus, the improved performance and strengthened brain networks in the intervention group could be explained by longer duration or the increased frequency of engagement. Although we cannot preclude this possibility, we nonetheless believe that the positive effects are at least partially a result of some specific components in the multimodal training. The intervention group showed improved memory and TMT performance, corresponding to the memory and executive function components in the cognitive training. The prefrontal cortex and MTL regions are also areas typically affected by EFT and memory strategy training (Valenzuela et al., 2003; Engvig et al., 2010; Belleville et al., 2011; Kirchhoff et al., 2012; Voss et al., 2012). Furthermore, the gains of cognitive function were correlated with the functional connectivity changes in these areas. Nevertheless, it would be ideal for the control and intervention groups to be matched in intervention frequency, duration, and even nature in future studies. Second, the fact that we did not randomly allocate individual participants to the intervention and control groups harms the validity of the research because of potential sampling bias. Although no significant group difference was found in the baseline characteristics, we cannot rule out systematic differences between the two groups. The reason we recruited these two groups from two similar communities, respectively, was to control expectancy effects. All participants were blind to the experiment design. If we had allocated participants from the same community to the intervention and control groups, then there would have been a high likelihood of the participants discovering the other group’s intervention contents through daily communications, and potentially guessing the study’s design and purpose. The non-randomized design may also have attrition bias. Four participants in the intervention group withdrew due to scheduling conflicts before the first intervention session, while there was no dropout at this phase in the control group; however, the dropout rates were equivalent in the two groups during the intervention phase. Third, using a two-group comparison hinders clarification of the contribution of each specific component (cognitive training, Tai Chi, and group counseling) and their interactions in the multimodal intervention. Fourth, we did not assess the long-term effects of the intervention on resting-state connectivity. Further studies are needed to examine whether the intervention-induced brain changes are maintained over time. In addition, we still do not fully understand the neural mechanisms underlying the plasticity of resting-state connectivity. However, combining multimodal neuroimaging data can be helpful for investigating the structure-function relationship of such plasticity. Moreover, extending this study to include other resting-state networks and whole-brain connectivity will also be an interesting and important complement to the current investigation of plasticity in older adults.

In summary, we have demonstrated enhanced resting-state connectivity between the mPFC and MTL regions following a multimodal intervention in older adults. Furthermore, the strength of mPFC-MTL connectivity after intervention predicted individual variability in neuropsychological performance. Our results provide evidence that brain regions greatly affected by aging may retain remarkable plasticity, and that this plasticity may be a crucial factor for helping older individuals maintain a healthy brain.

## Conflict of Interest Statement

The authors declare that the research was conducted in the absence of any commercial or financial relationships that could be construed as a potential conflict of interest.

## Footnotes

1 http://www.fil.ion.ucl.ac.uk/spm

2 http://restfmri.net

3 http://www.restfmri.net
